# Analysis of the Feasibility of the OrthoNail Hybrid Intramedullary Implant in the Human Body with Respect to Material Durability

**DOI:** 10.3390/jfb16010027

**Published:** 2025-01-15

**Authors:** Dominika Grygier, Piotr Kowalewski, Mariusz Opałka, Jakub J. Słowiński, Mateusz Dziubek, Dariusz Pyka

**Affiliations:** 1 Department of Vehicle Engineering, Faculty of Mechanical Engineering, Wroclaw University of Science and Technology, Smoluchowskiego 25 Str., 50-370 Wroclaw, Poland; dominika.grygier@pwr.edu.pl (D.G.); mateusz.dziubek@pwr.edu.pl (M.D.); 2 Orthoget Sp. z o.o., Muchoborska 18, 54-424 Wrocław, Poland; 3 Department of Fundamentals of Machine Design and Mechatronic Systems, Faculty of Mechanical Engineering, Wroclaw University of Science and Technology, Łukasiewicza 5, 50-371 Wroclaw, Poland; piotr.kowalewski@pwr.edu.pl (P.K.); mariusz.opalka@pwr.edu.pl (M.O.); 4 Department of Mechanics, Materials and Biomedical Engineering, Faculty of Mechanical Engineering, Wrocław University of Science and Technology, Smoluchowskiego 25, 50-372 Wrocław, Poland; dariusz.pyka@pwr.edu.pl

**Keywords:** OrthoNail, intramedullary implant, bone lengthening, mechanical testing, material durability

## Abstract

This study focuses on the development and evaluation of the OrthoNail hybrid intramedullary implant for lower limb lengthening in patients requiring significant skeletal reconstruction. The implant addresses the challenges in load-bearing during rehabilitation, providing a robust solution that is capable of supporting physiological loads. Mechanical tests, including axial compression, tension, torsion, and 3,4-point bending, determined the implant’s load capacity and fatigue resistance, while finite element analysis assessed stress distributions in bone tissue and around screw holes during single-leg stance, with boundary conditions derived from Orthoload database data. The OrthoNail implant demonstrated excellent mechanical stability, sustaining torsional loads of up to 19.36 Nm at maximum elongation (80 mm) and 17.16 Nm at zero elongation. Under axial compression, it withstood forces of up to 1400 N, maintaining structural integrity. Fatigue testing revealed resilience under dynamic loading conditions for over 1,000,000 cycles at a load of 500 N, with no mechanical failure or material degradation observed. Stress concentrations near screw holes indicate areas for potential optimization. The findings indicate that the OrthoNail implant demonstrates excellent mechanical stability and is well-suited for clinical application, enabling early full weight-bearing during rehabilitation.

## 1. Introduction

Implants are designed to restore functionality, enhance capabilities, or introduce new functions to the human body. The spectrum of implants is continuously expanding, driven by technological advances in fields such as bioengineering and medicine. Personalized treatment can dramatically improve patients’ quality of life, enabling therapies that would have been unachievable just decades ago [1,2,3,4]. This approach ensures better therapeutic outcomes, increased patient comfort, and a reduced risk of complications associated with mismatches between the implant and anatomical or biomechanical conditions.

The main purpose of a lower limb bone implant is to stabilize bone fragments and support load transfer through the kinematic chain [5]. Implants used in the lower extremities must withstand stresses arising from both the patient’s body weight and the intensity of physical activity, resulting in higher strength requirements than for implants used in other parts of the body [6]. The lower limbs must bear the loads associated with walking, running, and jumping and the dynamic force changes accompanying daily activities. Beyond fracture stabilization, these implants can also be used for osteosynthesis to increase bone length, thereby lengthening the limb.

Lower limb lengthening using intramedullary nailing has been performed for several decades. This method represents the most advanced evolution of the technique proposed by Ilizarov in the mid-20th century [7], which involved osteosynthesis using an external ring system. The Ilizarov method, while more affordable, significantly restricts patient mobility and comfort, is associated with considerable pain, and necessitates maintaining the highest standards of sterility throughout the treatment. Additionally, this method often leaves permanent skin scars. Intramedullary implants currently used for lengthening differ in terms of design, drive mechanism, material, and load-bearing capacity.

A significant limitation to date has been the ability of these implants to bear only partial loads resulting from the patient’s weight and activity level. Consequently, this limitation has restricted patient mobility and affected overall treatment comfort. The proposed OrthoNail hybrid-driven intramedullary implant design represents a significant advancement in this technology. Among the metallic biomaterials widely used in modern implantology, titanium alloys, particularly Ti-6Al-4V, are exemplary. This alloy was selected as the base material for the OrthoNail implant due to its excellent biocompatibility, high corrosion resistance, and osteointegration potential [8,9], which are crucial for successful implantation and achieving permanent integration with the surrounding bone tissue.

The appropriate selection of material properties also ensures optimal mobility in the fracture region. Micro-movements within the fracture gap, typically ranging between 50 and 200 μm, serve as a stimulus for bone tissue reconstruction by promoting cellular activity and mechanical signaling. This concept has been recognized for years as an essential factor in promoting bone healing and fusion [10,11].

Over the past two decades, the number of procedures related to the broadly understood fields of aesthetic medicine and surgery has significantly increased [12]. This trend is reflected in the growing number of scientific studies and publications examining various aspects of these practices. Aesthetic medicine encompasses procedures of varying complexity, addressing facial correction, dental aesthetics, hair restoration, scar removal, the treatment of various skin defects, fat reduction, and even height correction [13,14,15].

Trends indicating patient interest and the development of techniques by specialists are now highly pronounced. Certain geographical regions have specialized in specific medical procedures, with local practitioners focusing on particular treatments [16,17]. The age of patients opting for such procedures has decreased, and, with increased awareness and accessibility, the financial availability of these medical services has also risen [18]. The widespread presence of social media and the trends that they promote have undeniably influenced the growing interest in these types of medical interventions [19]. Patients who have undergone such treatments often report positive effects on their personal and professional lives [20,21].

However, the ethical aspect of these procedures cannot be overlooked. Physicians must prioritize the well-being of their patients and, in justified cases, should dissuade them from undergoing specific aesthetic procedures that could significantly impact their future lives [22,23]. All these phenomena fall under the broad definition of medical tourism.

Any intervention in the patient’s body inherently carries a risk of failure, which must be minimized at all costs. The current state of medical science nearly guarantees the elimination of risks known just a few decades ago. However, alongside the improvement in treatment quality, patient expectations and demands have increased, leading to entirely new areas of potential risk. Addressing and mitigating these risks is a natural step in the field’s evolution. The solution that we propose considers certain existing risks and offers methods for their minimization.

Intramedullary implants designed for lengthening long bones (particularly in the lower limbs) differ significantly from those used in stabilizing fractures of long bones. The primary challenge for these devices lies in the requirement to partially bear loads associated with basic patient locomotion. Due to the necessity of incorporating a drive mechanism, space must be allocated for these components, which is achieved at the expense of material, leading to reduced mechanical strength. Consequently, these implants typically have a larger diameter, and their load-bearing capacity is limited. This structural characteristic results in one of the primary recommendations given to patients: learning to partially weight-bear on the treated limb. Commonly, the recommendation is to load the implanted limb at approximately 20% of its nominal capacity. For this reason, patients in the early stages of treatment use crutches, walkers, or wheelchairs, especially in cases of bilateral implantation.

Among the existing solutions, the leading device is the Precice intramedullary implant manufactured by NuVasive Inc. According to the manufacturer’s recommendations, weight-bearing during the lengthening phase is only permissible within a limited range. Based on the operative technique provided by the manufacturer, the permissible load for implants of given diameters is as follows:

- 13.6 kg (30 lbs) for an implant with a diameter of 8.5 mm;
- 22.7 kg (50 lbs) for an implant with a diameter of 10.7 mm;
- 22.7 kg (50 lbs) for an implant with a diameter of 12.5 mm.

Only after the consolidation of bone fragments covering 75% of the cortical circumference in the osteotomy area can the limb load be further increased [24]. In many studies on the lengthening process using the Precice implant, the maximum load allowed during the lengthening phase was set at 20.0 kg [25,26]. Despite improvements introduced in subsequent versions, such as Precice 2, the achievable load capacity during the lengthening phase increased only to 34.0 kg (75 lbs) for a 12.5 mm diameter implant [27].

Research highlights the importance of adhering to recommendations for partial weight-bearing in the early stages of treatment. Overloading the implant before full bone consolidation can lead to implant material failure, necessitating surgical intervention to remove and replace the damaged component. To minimize the risk of complications such as implant failure, bone non-union, or soft tissue damage, patients must strictly adhere to weight-bearing limits, especially during the initial weeks after surgery.

Implant overload can cause material failure, requiring its removal and, if possible, replacement. The most commonly used tools for this procedure include various types of pliers and hooks, particularly useful for extracting the distal segment of the implant. Cannulated implants used for stabilizing long bone fractures are easier to extract due to multiple anchoring points [28]. In contrast, limb-lengthening implants are solid, which complicates their extraction [29]. Additionally, these implants contain components that are responsible for lengthening. If the implant fails at the drive mechanism, the resulting fragments may be numerous and difficult to remove due to their size and location.

A 2020 study described the first clinical case of a 20-year-old patient in whom a Precice implant fractured at the magnetic drive after a femoral lengthening of 5.3 cm. Implant failure dividing it into two parts poses significant challenges in extracting the distal portion, primarily due to the lack of specialized instruments [30]. To minimize the risk of implant fracture, strict adherence to weight-bearing guidelines during the consolidation phase is essential. Alternatively, using implants with larger diameters to provide greater strength can reduce fracture risk [30]. However, this approach is viable only for patients with sufficient intramedullary canal diameter. A common practice is to select an implant of the maximum possible diameter to enable the transfer of greater loads.

During limb-lengthening procedures, situations may arise where further distraction is impossible. Contributing factors may include increased resistance from soft tissues, joint contractures, or subluxations and dislocations. Additional risks are neurovascular injury, infections, poor regenerative potential, and premature consolidation. Eltayeby et al. introduced the concept of so-called dormant implants, which can remain in the patient’s body until the inhibiting factors are resolved [31]. This approach was based on findings indicating that nearly 85% of implants removed after treatment showed no signs of mechanical wear.

In one case, tibial lengthening was initially performed to achieve 4 cm. Due to contractures, the procedure was paused for two years, allowing for full bone consolidation and patient rehabilitation to enable further treatment. A re-osteotomy was then performed, achieving an additional 2 cm of lengthening. Unfortunately, the implant fractured at the interfragmentary gap, requiring removal. During extraction, the implant broke further, requiring removal piece by piece. The authors suggested that the implant likely failed due to overload occurring during the patient’s rehabilitation. Despite the complications, the bone fragments consolidated within a year. The authors concluded that using dormant implants carries significant risks but may be feasible with thorough pre-treatment evaluation [32].

It is important to note that implant failure is always a hazardous event that may damage tissues, preventing the planned lengthening and potentially leading to patient disability. Therefore, the primary goal is to develop implant designs that minimize such risks while increasing the load capacity of the implant without compromising its integrity.

The OrthoNail implant, currently in clinical trials, has been specifically designed for young, active patients. Constructed using the well-established Ti-6Al-4V alloy, its design allows it to bear loads equivalent to 80% of the body weight of a 90.0 kg patient. This feature enhances mobility and accelerates the recovery of functional capacity. From the patient’s perspective, a key advantage is the potential for a faster return to normal daily life and the restoration of independence. Additionally, the implant’s increased mechanical strength significantly reduces the risk of failure due to overloading, further ensuring its reliability and safety.

This study aimed to evaluate the mechanical strength of the OrthoNail hybrid intramedullary implant under typical physical loads. It assessed whether the implant’s design and materials can withstand increasing loads during rehabilitation, ensuring safe use in high-load-bearing procedures. By reducing complications like material failure, the implant supports full functionality during daily activities and intensive tasks. This enhances the post-operative quality of life.

The null hypothesis of this study posits that the OrthoNail hybrid intramedullary implant has sufficient mechanical strength and performance. It is designed to meet the demands of daily activities and postoperative rehabilitation, ensuring both safety and functionality during use.

## 2. Materials and Methods

The study focused on the OrthoNail hybrid intramedullary implant, designed for lower-limb lengthening with a maximum extension of 80 mm. The implant was analyzed in three configurations: minimal extension, partial extension (40 mm), and maximum extension (80 mm), to assess the effect of extension length on load-deformation characteristics and the feasibility of full weight-bearing.

As part of the mechanical testing, the mechanical parameters of the implant were evaluated under load conditions approximating normal use, specifically at 80% of the implant’s nominal load capacity.

Strength testing included compression, tensile, bending, and torsion tests conducted at various degrees of extension, as well as fatigue tests performed in accordance with ASTM and PN-EN standards. The following tests were conducted:

- Material testing (structure, hardness, and strength of the material);
- Testing on a proprietary strength testing setup, including the following:
  – Compression testing;
  – Tensile testing;
  – 3-point bending in frontal and sagittal planes;
  – Maximum load testing in compression, tension, and 3-point bending;
- Fatigue testing in 3-point bending;
- Static 4-point bending test;
- Static torsion test;
- Dynamic 4-point bending test;
- Finite element method (FEM) analysis of stress distribution in bone tissue at the site of bone screw insertion.

### 2.1. Implant Material Testing

The material used for the production of the OrthoNail implant is the titanium alloy Ti-6Al-4V ELI Heat No. PVD8154, conforming to ISO 5832-3:2021 [33]. The microstructure of the OrthoNail implant components was evaluated using a Nikon Eclipse MA200 light microscope (Nikon Corporation, Tokyo, Japan). Observations were performed on both unetched and etched samples at magnifications ranging from ×100 to ×1000. Images were captured using a Visitron Systems digital camera integrated with the microscope. Spot Advanced (v.5.6) and NIS Elements BR software (v.6.02.00) were used for image processing. Metallographic specimens were prepared in longitudinal and transverse directions relative to the axis of the element. Preparation involved mechanical grinding, polishing, and chemical etching with 10% HF.

Hardness measurements were conducted on cross-sections using the Vickers method, in compliance with the PN-EN ISO 6507-1:2018 standard [34]. The measurements were performed with a Matsuzawa MMT-X microhardness tester (Matsuzawa Co., Ltd., Akita, Japan).

To determine the material’s mechanical properties, static tensile tests were carried out according to PN-EN-ISO-6892-1:2019 [35]. The tests were conducted at room temperature using an FPZ 100/1 testing machine. During the static tensile test, the elongation was measured as a function of the applied force until sample rupture. The testing machine used had a force range of 0÷100kN.

### 2.2. Implant Strength Testing

#### 2.2.1. Testing on a Custom Strength Testing Setup

**Figure 1:**
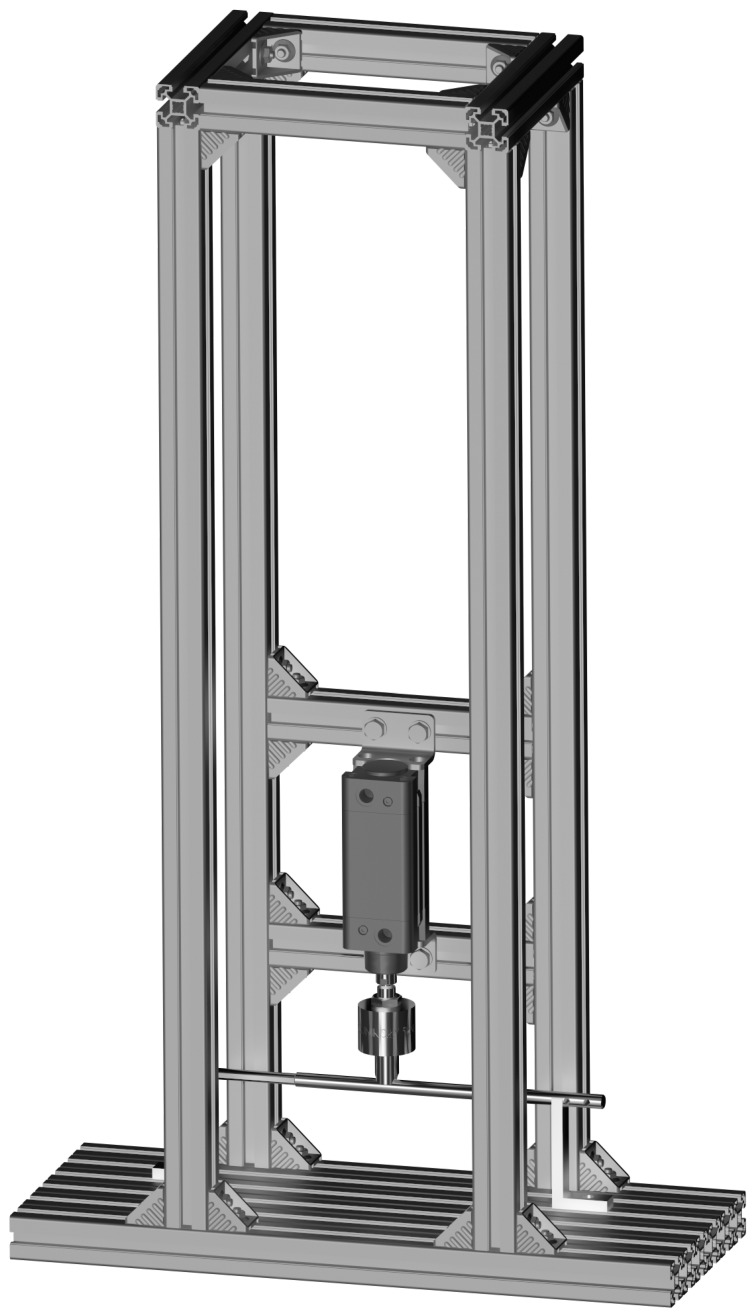
Custom strength testing setup developed for the study.

To conduct bending, compression, and tensile tests on the implant, a modular strength testing setup was constructed—Figure 1. The load-bearing structure of the setup was built using Bosch-Rexroth 40 × 40 aluminum profiles. A pneumatic actuator (blue) with a piston diameter of 50 mm and a stroke of 50 mm, operating at a maximum pressure of 8 bar, was used as the force-generating component. Mounting fixtures (yellow) used in the experiments were fabricated from heat-treated structural steel grade S355J0. The design of the mounting fixtures and the method of securing the intramedullary implant were developed based on testing procedures described in the literature [36,37].

For measurements, strain gauge force sensors (green) and a precision laser displacement sensor with a measurement range of 100 mm were employed. The tests aimed to determine the load-deformation characteristics of the implant prototype (intramedullary implant). The following loading scenarios were applied:

- Compression in the range $F_{c}=0÷500\;N$;
- Tension in the range $F_{t}=0÷100\;N$;
- 3-point bending with a bending moment range $M_{b}=0÷25\;\mathrm{Nm}$ (in both frontal and sagittal planes).

The tests were conducted during both loading and unloading cycles, with a minimum of six repetitions for three different implant extension lengths (fully retracted, half-extended (40 mm), and fully-extended (80 mm)). The number of repetitions for various implant configurations, including extension levels and geometric planes, is schematically summarized in Table 1.

For configurations where the implant demonstrated the lowest stiffness (i.e., maximum extension), additional load-deformation tests were performed under the maximum loads achievable with the constructed testing setup.

#### 2.2.2. Compression Test

The compression test was conducted by subjecting the implant to axial compressive loading, applied through the displacement of the piston in the upper traverse of the custom-built strength testing setup. The load was applied at a rate of approximately $10\;{\mathrm{Ns}}^{-1}$. The test was performed under controlled temperature conditions (23 ± 5 °C). During the test, the load and displacement of the upper traverse were recorded. The test was continued until the maximum force specified in the experimental plan, i.e., 500 N, was reached, or until permanent deformation or damage preventing the further continuation of the test occurred.

The test was performed for three implant configurations: fully retracted (0 mm), half extended (40 mm), and fully extended (80 mm). After each test, the intramedullary implant was subjected to a visual inspection (in accordance with PN-EN 13018:2016-04 [38]) to identify potential defects caused by the applied load.

#### 2.2.3. Tensile Test

The tensile test was conducted by subjecting the implant to axial tensile loading, applied through the retraction of the piston in the upper traverse of the strength testing setup. The load was applied at a rate of approximately $10\;{\mathrm{Ns}}^{-1}$. The test was performed under controlled temperature conditions (23 ± 5 °C). During the test, the load and displacement of the upper traverse were recorded. The test was continued until the maximum force specified in the experimental plan, i.e., 100 N, was reached, or until permanent deformation or damage preventing further continuation of the test occurred.

The test was performed for three implant configurations: fully retracted, extended to 40 mm, and fully extended. After each test, the intramedullary implant was subjected to a visual inspection (in accordance with PN-EN 13018:2016-04 [38]) to identify potential defects caused by the applied load.

#### 2.2.4. Bending Test

The bending test was conducted by subjecting the implant to axial bending loads, applied through the displacement of the piston in the upper traverse of the strength testing setup. The 3-point bending test was performed following the PN-EN ISO 7438:2021:04 [39] standard, with loading applied in a single direction until the planned bending moment was reached. The test was carried out under controlled temperature conditions (23 ± 5 °C). The load was applied at a rate of approximately $10\;{\mathrm{Ns}}^{-1}$, allowing for the potential plastic deformation of the material. During the test, the load and the deflection of the implant were recorded.

The test was continued until the maximum bending moment specified in the experimental plan, i.e., 25 Nm, was reached, or until permanent deformation or damage preventing further continuation of the test occurred. The test was performed for three implant configurations: fully retracted, extended to 40 mm, and fully extended. For each configuration, tests were conducted for both the frontal and sagittal planes of the implant.

The bending test for the three extension positions required adjustments to the span between supports, which were set at 250 mm, 290 mm, and 315 mm, respectively. The span remained constant during each individual test. The distances between supports were selected based on the degree of implant extension.

After each test, the intramedullary implant was subjected to a visual inspection (in accordance with PN-EN 13018:2016-04 [38]) to identify potential defects caused by the applied load. This inspection served as the compliance evaluation criterion for the product.

#### 2.2.5. Fatigue Testing in 3-Point Bending

**Figure 2:**
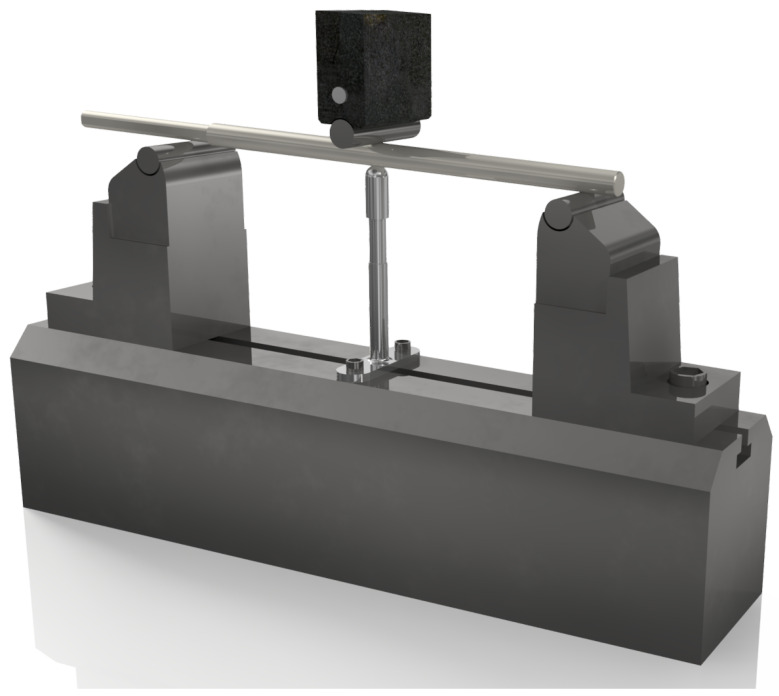
General view of an example implant prepared for fatigue testing in the 3-point bending configuration.

The fatigue test conducted on a custom-built testing setup allowed for the determination of deflection changes during the fatigue process after 500,000 load cycles. The setup was configured as a 3-support system with the following parameters: support span $d_{1}=250\;\mathrm{mm}$, pin diameter $d_{2}=10\;\mathrm{mm}$, and support diameter $d_{3}=10\;\mathrm{mm}$—Figure 2.

The loading was applied using an Instron XYZ testing machine under the following conditions:

- Mean bending force (The mean value was calculated for cycles in the range of 100 to 500,000, after stabilization of the recorded results): $F_{\mathrm{mean}}=-158.19\pm 0.25\;N$;
- Mean bending moment: $M_{\mathrm{mean}}=-9.89\pm 0.02\;\mathrm{Nm}$;
- Mean deflection: $f_{\mathrm{mean}}=-0.71\pm 0.02\;\mathrm{mm}$.

The test was performed on the implant at its maximum extension, i.e., 80 mm. The implant was positioned and loaded in the frontal plane within the testing machine. Data collection during the test was performed according to the scheme outlined in Table 2.

#### 2.2.6. Static 4-Point Bending Test

As part of the strength analysis of the OrthoNail hybrid intramedullary implant, a static 4-point bending test was conducted in accordance with the ASTM F1264-16ϵ1 standard [40]. This test is a standard evaluation tool for implants designed for bone stabilization, providing precise information on the stiffness and resistance of the material to bending moments that the implant may experience during normal patient activities.

**Figure 3:**
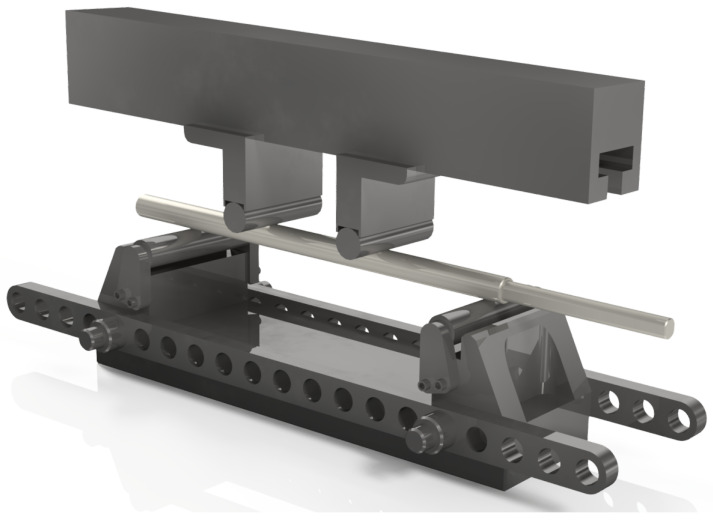
Positioning of the implant on supports during the 4-point bending test.

**Figure 4:**
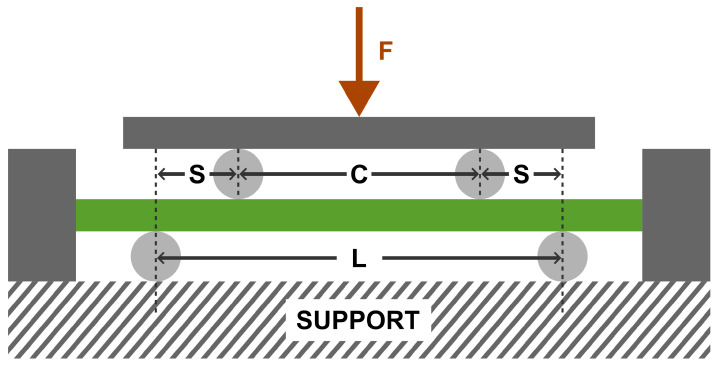
The 4-Point bending schematic [40].

The test was performed using an MTS Criterion Model 43 testing setup (Figure 3), with the implant positioned on supports spaced at 76 mm (inner span) and 228 mm (outer span). The loading and supporting rollers had a diameter of 20 mm, ensuring even force distribution (Figure 4). The load was applied at a displacement rate of 1 mm/s, and the test continued until the maximum load for the structure was reached or deformation occurred that disqualified the implant. This test was conducted on two implants: one at zero elongation (0 mm) and the other at the maximum elongation of the implant (80 mm).

During the test, the force-displacement relationship was recorded. Additionally, the load corresponding to a 0.2% displacement offset $F_{y}$, stiffness ($EI_{e}$), and bending moment ($M_{y}$) were calculated based on the following relationships ($D_{\mathrm{IMFD}}$—implant diameter):

(1) $$y_{0.2\%}=\frac{S(L+2C)}{\left(1500\times D_{\mathrm{IMFD}}\right)}$$

(2) $$M_{y}=\frac{F_{y}S}{2}$$

(3) $$EI_{e}=\frac{s^{2}\left(L+2C\right)\left(F/y\right)}{12}$$

#### 2.2.7. Static Torsion Test

To investigate the mechanical strength of the OrthoNail hybrid intramedullary implant, a static torsion test was conducted. This test, performed in accordance with ASTM guidelines [40], allowed the evaluation of the material and structural resistance to torsional moments that the implant may encounter during daily activities, potentially increasing the risk of deformation or material decohesion under clinical conditions.

**Figure 5:**
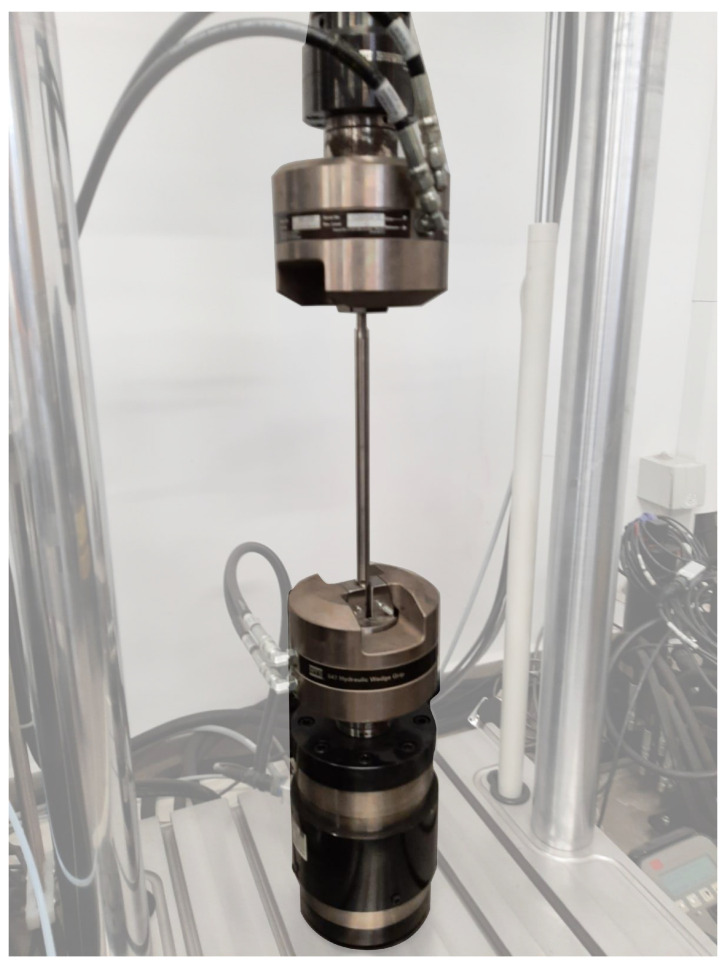
Implant positioning in clamps during the static torsion test.

During the test, the implant was secured in the pneumatic grips of an MTS Bionix testing machine, maintaining a constant embedding depth (25 mm) and a distance between grips of 230 mm (Figure 5). The test was carried out at a rotational speed of 5°/min until the occurrence of disqualifying deformation or material failure. This test was conducted on two implants: one at zero elongation (0 mm) and the other at the maximum elongation of the implant (80 mm).

#### 2.2.8. Dynamic 4-Point Bending Test

The dynamic 4-point bending test was performed to assess the resistance of the OrthoNail hybrid implant to cyclic loads, simulating real-world conditions that may occur during a patient’s daily activities and rehabilitation. This test is crucial, as it determines the long-term stability and fatigue wear resistance of the material and design.

The tests were conducted on a setup equipped with supporting and loading rollers with a diameter of 20 mm and support spans of S=C=76mm and L=228mm, exactly the same as in the static test. Two load values were used during testing. The first load value was determined according to standard guidelines [40]. Based on the results of the static 4-point bending test, the maximum destructive force was determined. For fatigue testing, 75% of the maximum force value from the static tests was applied as the maximum load. According to material failure mechanics [41], 75% of $F_{\max}$ represents a value where the specimen operates within the elastic strain range. The minimum load $F_{\min}$ was set at 10% of the maximum force to meet the condition specified in the standard [40], where R=0.1. The load followed a sinusoidal pattern from $F_{\min}$ to $F_{\max}$. The tests were conducted at a frequency of 5Hz on two implants: one at zero elongation (0 mm) and the other at the maximum elongation of the implant (80 mm).

The applied methodology is utilized for comparative evaluation of implants with different designs, materials, and sizes and is not intended for product assessment in clinical applications. The second load value was selected to simulate physiological loads experienced in the anatomical system. The load values were derived from literature data [42,43,44,45,46,47]. For an active patient, the bending load on the femur reaches 1000 N. The minimum load $F_{\min}$ was set at 100 N, while the maximum load $F_{\max}$ was 1000 N. The adopted load ratio ensured compliance with the R=0.1, as defined in the standard [40].

During the conducted tests, the number of working cycles for the implants was recorded. For the first type of loading, the number of cycles was registered until the test specimen failed, whereas, for the second type of loading, it was checked whether the specimen would fail before reaching 1 million (10^6^) cycles. The number of cycles was selected based on normative requirements [40], which assume that the healing time of a fracture stabilized with an intramedullary implant lasts approximately 3 months. This period corresponds to about 150,000 to 250,000 cycles. Therefore, fatigue resistance at 10^6^ cycles significantly exceeds the expected clinical demand for this type of structure.

### 2.3. Numerical Calculations

A numerical analysis was conducted in this study to evaluate the stress distribution in bone tissue in the region of screw holes connecting the implant with the tissue. This analysis aimed not only to investigate stress distribution but also to validate the mechanical stability of the OrthoNail implant under physiological loading conditions. Such studies are essential for optimizing implant designs and reducing the risk of mechanical failure in clinical applications.

**Figure 6:**
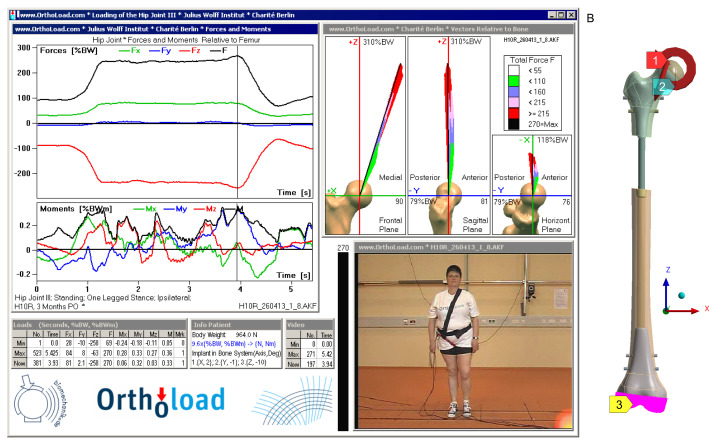
Boundary conditions used in simulation: (**A**) load on the femur resulting from one-legged stance [48], (**B**) developed numerical model of the femur with applied conditions: 1—applied force, 2—applied moment, 3—fixation.

The loading conditions were based on a one-legged stance, which represents a typical and clinically relevant scenario for evaluating implant performance. The patient’s body weight, taken from a study conducted at the Julius Wolff Institute, was 98.0 kg. Force and moment values were adopted from the Orthoload database (file h10r_260413_1_8), corresponding to the time point t=3.93s, where these parameters reached their maximum values. This time point represents the worst-case loading scenario during the stance phase. The force and moment were applied to the femoral head, while the distal femoral epiphysis was fixed by constraining all degrees of freedom, as shown in Figure 6.

The geometric model was created based on a commercially available geometric model of the femur provided by Sawbones. An osteotomy was introduced in the bone model, and the fragments were separated by a distance of 80 mm, corresponding to maximal implant extension. The OrthoNail implant was inserted into the model, replicating the expected surgical scenario.

The model was meshed using approximately 315,000 higher-order tetrahedral finite elements, with a refined element size of 1 mm around critical regions such as the screw holes. A mesh convergence study was conducted to ensure that the results were independent of the mesh density. The material properties used in the model are presented in Table 3. The developed model assumed isotropic material properties for both cortical and cancellous bone, which simplifies the analysis but may underestimate the influence of anisotropy, particularly in areas of high stress concentration.

The contact interactions between the implant and the surrounding tissue were modeled as frictional contact (μ=0.38), while the connections between the screws and the tissue were assumed to be bonded contact.

## 3. Results

### 3.1. Implant Material Analysis

**Figure 7:**
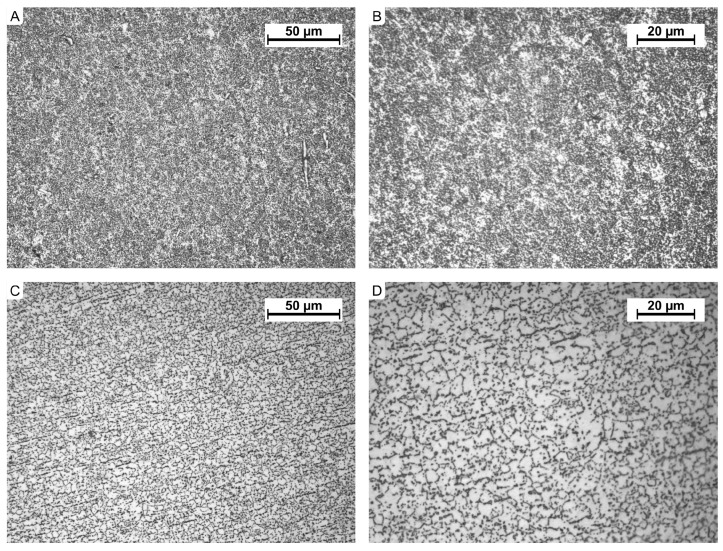
Microstructure of Ti-6Al-4V alloy: (**A**) etched with 10% HF, transverse section, magnification ×500, (**B**) etched with 10% HF, transverse section, magnification ×1000, (**C**) etched with 10% HF, longitudinal section, magnification ×500, (**D**) etched with 10% HF, longitudinal section, magnification ×1000.

A microscopic examination of the material revealed the presence of an (α+β) structure containing approximately 20% of the α phase. The size of the precipitates complies with the A1 standard according to ISO 20160 (Figure 7).

The material exhibits a hardness of approximately 301 HV (Table 4), tensile strength of 1061 MPa, and yield strength of 905 MPa. The mechanical properties (Table 5) conform to ISO 5832-3:2021 [33] standards and the manufacturer’s certification.

The obtained strength test results indicate that the OrthoNail hybrid implant exhibits high resistance to mechanical loads in axial compression, tension, and bending and torsion tests. This robustness is crucial for ensuring its stability and functionality during daily use by patients.

### 3.2. Mechanical Strength Testing of the Implant

#### 3.2.1. Testing on a Custom Strength Testing Rig

Table 6 presents the averaged results obtained during the determination of the implant’s load-deformation characteristics. The table includes the mean value of the recorded force or bending moment along with the standard deviation for six repetitions in each specific implant configuration. Additionally, for the maximum recorded load values, the corresponding maximum displacement values were measured and averaged for each case, as shown in Table 6.

Based on the conducted tests, it can be concluded that the prototype intramedullary implant demonstrates mechanical strength within the following ranges:

- Axial compression: 500 N;
- Axial tension: 100 N;
- Bending: 20 Nm (in both frontal and sagittal planes).

Additionally, testing under maximum load conditions demonstrated the strength of the fully extended intramedullary implant prototype in the following ranges:

- Axial compression: 1400 N;
- Axial tension: 1000 N;
- Bending: 112–115 Nm (in both frontal and sagittal planes).

In axial compression tests conducted within a force range of 0 to 500 N, the implant exhibited structural stability in various extension positions (fully retracted, 40 mm extended, and fully extended to 80 mm). In axial tension tests conducted within a force range of 0 to 100 N, the implant demonstrated resistance to tensile loads, which is particularly critical for its durability during movements requiring material flexibility. The averaged results from these tests indicate complete mechanical stability, confirming that the OrthoNail implant can effectively support full loading during the rehabilitation phase without deformation risk.

The 3-point bending tests, conducted in both the frontal and sagittal planes, revealed significant resistance to bending moments up to 25 Nm in various extension configurations. The results from tests with different support spans (250 mm, 290 mm, 315 mm) confirm that the OrthoNail implant effectively transfers bending forces, which is crucial for stabilizing bone fragments during movements and dynamic loading.

#### 3.2.2. Fatigue Testing in 3-Point Bending

The results obtained during the complete course of the test (Table 7) revealed a deflection change of 1.43%, which did not exceed the predefined threshold of 10%. A visual inspection of the implant after testing showed no signs of damage, wear, or fractures that would indicate material fatigue.

Microscopic observations of the implant surfaces after strength tests were conducted using a 3D digital microscope (Keyence VHX-X1 Series) at magnifications ranging from ×20 to ×2000. The samples were prepared by cleaning the outer surface with soft wipes and acetone. The results of the observations did not indicate any material decohesion in the OrthoNail implant, confirming the high mechanical strength properties of the tested medical device.

#### 3.2.3. Static 4-Point Bending Test

The results of the static 4-point bending test for the OrthoNail implant met the expectations for implants with enhanced resistance to mechanical loads (Table 8). For the implant in the fully retracted position, a maximum force ($F_{\max}$ of 5.101 kN) was recorded, with a stiffness of 74 Nm^2^. For the fully extended configuration (80 mm), these values were 4.890 kN and 58 Nm^2^, respectively, indicating a decrease in stiffness at maximum extension while maintaining adequate structural strength to handle dynamic loads.

These results provide valuable insights into the implant’s resistance to bending forces that may act on the structure during daily activities such as walking or light physical exercises. The retention of strength at maximum extension confirms the implant’s ability to stably transfer loads even in the most demanding configurations, which is critical for the lower limb-lengthening process.

The test also demonstrated that the OrthoNail implant, despite reduced stiffness in the fully extended position, maintains structural integrity, minimizing the risk of permanent deformations and potential damage. Clinically, these results are significant as they confirm the feasibility of fully utilizing the implant during the later stages of rehabilitation when gradual increases in limb loading are required. For the patient, this translates to greater stability and safety during recovery, as well as a reduced risk of surgical interventions due to material failure or delamination.

The conclusions from the 4-point bending test indicate that the OrthoNail implant meets the requirements for resistance to dynamic loads and provides adequate stability for bone lengthening procedures. This can contribute to accelerating the treatment and rehabilitation process for patients following orthopedic surgeries.

#### 3.2.4. Static Torsion Test

The results of the torsion test showed that the OrthoNail implant achieved a maximum torsional moment of 17.16 Nm in the minimally extended position and 19.36 Nm at the maximum extension of 80 mm (Table 9). Although the greater extension was associated with a higher torsional moment, the implant maintained its structural integrity, demonstrating high resistance to torsional forces even in the most extended configuration. These results indicate the implant’s ability to transfer rotational forces without deformation risk, which is critical in bone-lengthening procedures where the implant may be subjected to such forces during rotational movements of the limb.

An analysis of the static torsion test results suggests that the OrthoNail implant has the potential to withstand torsional loads of up to over 19 Nm at maximum elongation, as observed in this study. These values align with those reported in previous works [6,48] (Orthoload—file k8l_280415_1_119p, level walking), indicating that the implant can safely accommodate loads during the rehabilitation phase. For patients, this translates to a reduced risk of implant failure during intense movements or rotational overloads, such as foot twists, which can occur during walking, climbing stairs, or performing basic physical exercises, as they gradually regain the ability to load and rotate the limb.

The conclusions from the static torsion test emphasize that the hybrid OrthoNail implant meets the high strength requirements, providing adequate stability and durability, which are necessary for bone-lengthening procedures. Its high resistance to torsional forces allows for the safe use of the implant during rehabilitation, contributing to a faster and more comfortable return to normal physical activity for patients post-surgery.

#### 3.2.5. Dynamic 4-Point Bending Test

**Figure 8:**
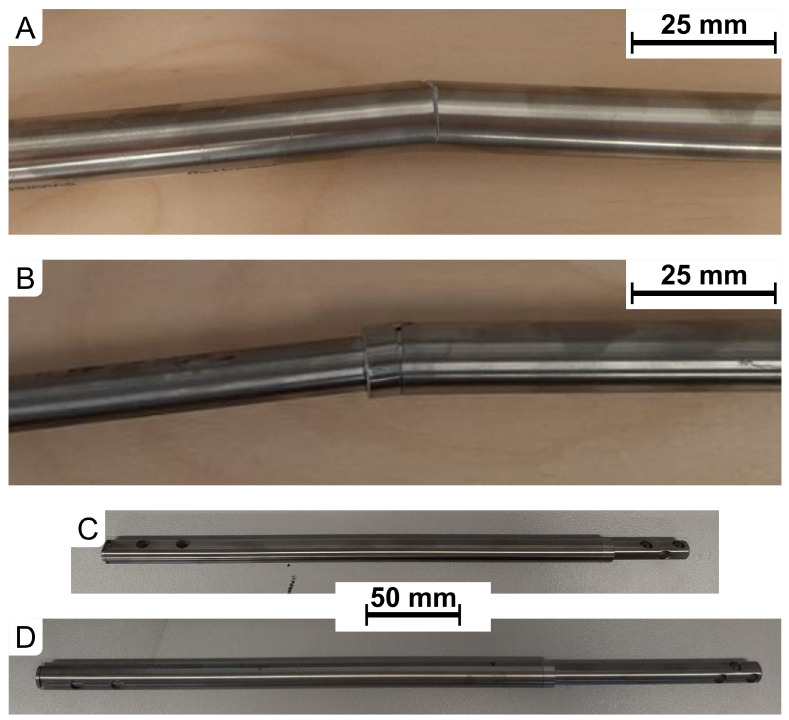
Condition of implants after completing the dynamic 4-point bending test: (**A**) (d=0mm, $F_{\max}=3900.0\;N$), (**B**) (d=80mm, $F_{\max}=2900.0\;N$), (**C**) (d=0mm, $F_{\max}=1000.0\;N$), (**D**) (d=80mm, $F_{\max}=1000.0\;N$).

Implants subjected to a load of 75% of $F_{\max}$ experienced material delamination, consistent with the assumptions of fatigue testing aimed at evaluating critical strength values (Table 10). In the fully retracted configuration (d=0mm), the implant fractured at a load of 3900 N, while, in the fully extended configuration (d=80mm), it fractured at 2900 N. The fractures occurred at different locations: for d=0mm, the failure was in the proximal section, whereas, for d=80mm, the failure occurred at the connection between the proximal and distal parts (Figure 8A,B). These results are typical for such designs exposed to significant bending moments. The findings confirm that, near the maximum strength load, there is a risk of material failure; however, the results align with the functional parameters expected for implants of this class.

Implants subjected to loads of 1000 N withstood the test without damage, even after 1 million cycles, which exceeds the anticipated clinical usage conditions. Following the test, visual inspection revealed no signs of wear or structural deformation (Figure 8C,D). The retention of structural integrity after prolonged cyclic loading indicates a high fatigue resistance of the OrthoNail implant, highlighting its potential for long-term use in patients’ daily activities without the risk of sudden failure.

The results of the dynamic 4-point bending test demonstrate that the OrthoNail implant has a high resistance to fatigue overload under conditions similar to physiological loads. The observed fatigue resistance confirms the durability of the implant, which can reduce the risk of requiring revision surgeries due to material failure. For patients, this translates to a quicker return to physical activities after surgery and a lower risk of complications related to implant damage during rehabilitation or extended use. These properties make the implant suitable for procedures requiring highly durable materials capable of withstanding prolonged cyclic loading, which is critical in orthopedics and trauma surgery.

In summary, the dynamic 4-point bending test confirmed that the hybrid OrthoNail implant is a stable and durable structure, making it a competitive option in the market for limb-lengthening implants. Its high resistance to dynamic loads can significantly contribute to improving patients’ quality of life by supporting their rehabilitation process and return to normal activities.

### 3.3. Numerical Analysis

**Figure 9:**
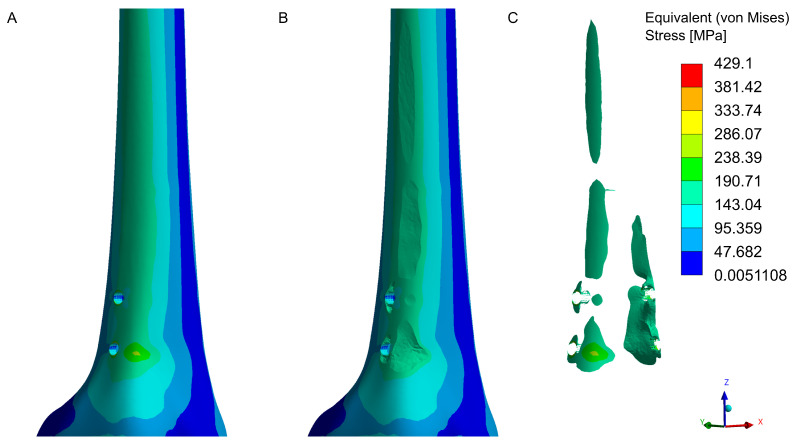

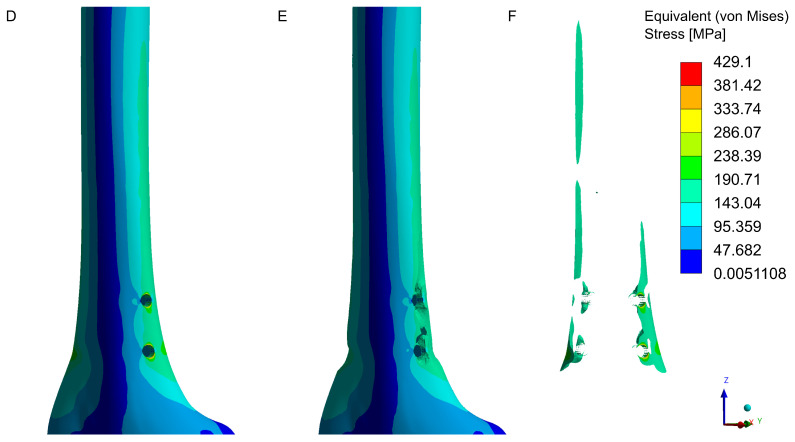
Equivalent (von Mises) stress concentration in the distal femoral fragment near the bone screw holes. Lateral view of the model: (**A**)—stress distribution in the complete model, (**B**)—model with elements exceeding 160 MPa removed, (**C**)—elements where stress exceeds 160 MPa. Medial view of the model: (**D**)—stress distribution in the complete model, (**E**)—model with elements exceeding 160 MPa removed, (**F**)—elements where stress exceeds 160 MPa.

The numerical analysis identified areas of maximum stress concentration near the screw holes. These zones indicate potential critical areas for bone tissue under high loads. The maximum stress in the model exceeded 160 MPa, surpassing the bone’s immediate compressive and tensile strength (Figure 9). Stress concentration areas were localized around the screw holes, with maximum values recorded on the edges of the holes and on the anterolateral and anteromedial surfaces. Stress concentration at sharp edges is a common phenomenon but can be reduced by introducing rounded edges. This process occurs naturally during drilling, as bone hole edges are never perfectly sharp, mitigating stress concentrations.

It is important to note that the developed model includes only bone tissue and the implant, without considering the supportive properties of soft tissues. Additionally, the presented model was created for a patient whose body weight exceeds the average for individuals undergoing such procedures, and the analyzed case involved the maximum designable extension of 80 mm. Typically, extensions are limited to around 50 mm due to the body’s regenerative capacity and the ability to extend soft tissues. Extensions beyond this threshold may increase the likelihood of soft tissue complications, impacting patient comfort and rehabilitation time.

A mesh sensitivity analysis revealed that refining the mesh in the screw hole areas to 0.3 mm does not lead to significant changes in the stress distribution within the model.

## 4. Discussion

Material and mechanical strength tests of the OrthoNail hybrid intramedullary implant confirmed its high mechanical resistance and fatigue durability under loading conditions representative of typical patient activities. These results validate the implant’s potential for clinical application in lower limb lengthening procedures. When compared to the existing literature and solutions, the findings reveal several notable distinctions.

In compression strength testing, the implant shell withstood a maximum load of 1400.0 N, meeting the requirement of transferring loads equivalent to 80% of the body weight of a 90.0 kg patient. Compared to the maximum load value suggested for the Precice implants, which is approximately 34.0 kg (about 334 N) during the lengthening phase [27], the OrthoNail implant significantly outperforms this design. Additionally, the maximum bending moment of 115 Nm confirms that OrthoNail can withstand bending forces typical of daily activities such as walking and light physical exercise. This is particularly important in increasing patient mobility and reducing the rehabilitation period. Eliminating the limitation of partial limb loading can significantly improve the patient’s quality of life. Moreover, an approach focused on early activation and mobilization of the patient is a commonly applied practice for patients undergoing surgical treatment, such as femoral fractures or planned total knee arthroplasty, which significantly shortens recovery time while yielding better outcomes [49,50].

The torsional moments for the OrthoNail implant, which reached values of up to over 19 Nm at maximum elongation, correspond with those reported in previous studies [6,48] (Orthoload—k8l_280415_1_119p). This suggests that such parameters are sufficient to safely accommodate loads, even during the rehabilitation phase.

The results also indicate that the OrthoNail implant maintains its structural integrity even at maximum extension, which is advantageous in procedures requiring maximum limb lengthening. However, it should be noted that the reduced stiffness at maximum extension, although expected for such designs, still provides biomechanical stability in the final stages of lengthening.

This structural stability enables the safe transfer of dynamic loads during rehabilitation, reducing the need for additional support or mobility aids, such as crutches or walkers, even in the early stages of treatment. These findings align with the reports by Eltayeby et al. [31], who highlighted the mechanical limitations of existing lengthening constructs.

Dynamic testing showed that the implant withstood loads of 1000 N for 1 million cycles, exceeding the typical clinical requirements (150,000–250,000 cycles) for the regeneration period following osteotomy [40]. This resistance is critical for the long-term use of the implant by patients, allowing safe limb loading at various stages of rehabilitation while minimizing the risk of complications related to fractures or material delamination. Clinically, this means that the OrthoNail implant can be used in patients’ daily activities for extended periods without requiring replacement.

The issue of implant resistance, particularly to bending loads, is significant in light of findings presented in studies [51,52]. The flexion of Precice implants used during femoral lengthening beyond 5° has been shown to cause damage to the lengthening mechanism, necessitating implant replacement, thereby impacting treatment quality. The bending mechanism in implants is driven by a significant discrepancy between the anatomical and mechanical axes of the femur. Therefore, the bending resistance of the OrthoNail implant could distinguish it from existing solutions.

Numerical analysis results indicate that stress concentration areas may appear at the sites where the implant is attached to the bone with screws. This is an expected effect and will depend on the type of activity and patient body weight. Consequently, weight reduction should be recommended before treatment if indicators such as BMI suggest it.

The Ti-6Al-4V alloy possesses structural and mechanical properties that comply with standards developed for medical implants, such as ASTM F136 [53] and ISO 5832-3:2021 [33]. Its application confirms its durability and suitability for the requirements of orthopedic use.

Further studies, as are necessary for a comprehensive evaluation of the implant, should focus on long-term clinical observations to assess the risk of delamination under actual-patient loading conditions. The impact of different extension configurations on biomechanical stability and patient comfort should also be analyzed. Furthermore, comparisons with other intramedullary implants under real clinical conditions are essential, including subjective patient opinions regarding comfort and treatment effectiveness.

The primary objective of this pilot study was to evaluate the mechanical parameters of the implant using a standards-compliant approach. As such, the inclusion of control groups or extensive statistical analyses was not initially planned. Nevertheless, all experimental procedures were conducted in strict accordance with the relevant standards, ensuring the highest level of rigor and precision. This methodology allowed for a thorough assessment of the implant’s mechanical properties while maintaining adherence to established testing protocols. However, the absence of control groups and extensive statistical analyses represents a limitation of this study, highlighting the need for future research with larger sample sizes, comparative testing, and broader statistical validation to further substantiate the findings.

Based on the obtained results and in reference to the available literature, the null hypothesis is considered valid. This conclusion is supported by the mechanical testing results, which demonstrated that certain parameters of our implant exceed those of competing solutions. However, it is important to emphasize that further testing is necessary, including large-sample studies, comparative tests with other existing solutions, and clinical trials. These additional investigations will help determine how the developed implant performs in bearing loads during bone-lengthening procedures and throughout the rehabilitation phase.

Conducting clinical trials on implants is crucial to determine the feasibility of using new materials in medical practice. Additionally, statistical studies are needed to evaluate the efficacy and safety of implants within the patient population.

## Figures and Tables

**Table 1:** Experimental scheme for mechanical testing of the implant.

Axial Compression of the Implant				
IL* = 0 mm		6 repetitions for $F_{c}=0÷500\;$ N for each implant lenght		
IL = 40 mm				
IL = 80 mm				
**Axial Tension of the Implant**				
IL = 0 mm		6 repetitions for $F_{c}=0÷500\;$ N for each implant lenght		
IL = 40 mm				
IL = 80 mm				
**3-Point Bending of the Implant**				
		Support Span		
		250 mm	290 mm	315 mm
IL = 0 mm	Frontal Plane	6 repetitions for $M_{b}=0÷25\;\mathrm{Nm}$		
	Sagittal Plane			
IL = 40 mm	Frontal Plane		6 repetitions for $M_{b}=0÷25\;\mathrm{Nm}$	
	Sagittal Plane			
IL = 80 mm	Frontal Plane			6 repetitions for $M_{b}=0÷25\;\mathrm{Nm}$
	Sagittal Plane			

* IL—implant length.

**Table 2:** Data recording method during fatigue testing in the 3-point bending configuration.

Cycles	Data Recording Frequency
1–10	Every cycle
11–100	Every 10 cycles
101–1000	Every 100 cycles
1001–500,000	Every 1000 cycles

**Table 3:** Material properties used in FEM analysis.

Material	E [GPa]	v [-]
Cortical bone tissue	18	0.3
Cancellous bone tissue	0.480	0.42
Titanium alloy	114	0.34

**Table 4:** Vickers hardness test results for the Ti-6Al-4V alloy.

No.	Hardness HV1 (9.807 N)	Mean Hardness HV1
1	296.5	301.00±4.95
2	300.3	
3	301.6	
4	299.3	
5	307.3	

**Table 5:** Static tensile test results for the Ti-6Al-4V alloy.

No.	Young’s Modulus	Elastic Limit	Yield Strength	Tensile Strength	Cross-Section Reduction	Elongation at Break
	E [$10^{5}$ MPa]	$R_{0.05}$ [MPa]	$R_{0.2}$ [MPa]	$R_{m}$ [MPa]	Z [%]	A [%]
1	1.10	772	792	1046	31	7.3
2	1.13	947	964	1051	34	9.3
3	1.13	944	959	1087	25	8.0
Average	1.12	888	905	1061	30	8.2

**Table 6:** Results obtained from custom strength testing rig measurements.

Axial Compression of the Implant				
		**Results (Load ± Displacement)**		
IL * = 0 mm		508.12 ± 0.91 N 0.24 ± 0.02 mm		
IL = 40 mm		511.53 ± 8.68 N 0.24 ± 0.03 mm		
IL = 80 mm		507.89 ± 2.09 N 0.49 ± 0.04 mm		
**Axial Tension of the Implant**				
IL = 0 mm		105.18 ± 0.76 N 0.05 ± 0.00 mm		
IL = 40 mm		104.27 ± 0.29 N 0.10 ± 0.01 mm		
IL = 80 mm		103.55 ± 0.82 N 0.07 ± 0.01 mm		
**3-Point Bending of the Implant**				
		**Support Span**		
		250 mm	290 mm	315 mm
IL = 0 mm	Frontal Plane	25.06 ± 0.03 Nm 1.55 ± 0.01 mm		
	Sagittal Plane	25.28 ± 0.29 Nm 1.56 ± 0.07 mm		
IL = 40 mm	Frontal Plane		25.35 ± 0.24 Nm 2.42 ± 0.04 mm	
	Sagittal Plane		25.18 ± 0.09 Nm 2.42 ± 0.03 mm	
IL = 80 mm	Frontal Plane			25.17 ± 0.04 Nm 3.03 ± 0.00 mm
	Sagittal Plane			25.25 ± 0.03 Nm 3.02 ± 0.03 mm

* IL—implant length.

**Table 7:** Fatigue test results for 3-point bending.

Parameter	Value	Unit
Maximum deflection for cycle 100	f(100)	−0.598568665 mm
Maximum deflection for cycle 500,000	f(500,000)	−0.590009205 mm
Deflection change	Δf	0.00855946 mm
Deflection change *	Δf	1.43%
Visual evaluation of implant post-test	no signs of damage	

* The change was calculated for cycles in the range of 100 to 500,000, after the obtained results had stabilized.

**Table 8:** Static 4-point bending test results.

Implant	$F_{\max}\;\left[\mathrm{kN}\right]$	*q*	$y_{0.2\%}\;\left[\mathrm{mm}\right]$	F[kN]	$F_{y}\;\left[\mathrm{kN}\right]$	y[mm]	${\mathrm{EI}}_{e}\;\left[{\mathrm{Nm}}^{2}\right]$
d=0mm	5.101	148.2	1.6	3.9	4.54	11.2	74
d=80mm	4.890	110.2	1.6	2.9	4.10	12.9	58

**Table 9:** Static torsion test results.

Implant Length	Torsional Moment [Nm]	Rotation Angle [°]
d=0mm	17.16	18.45
d=80mm	19.36	19.37

**Table 10:** Dynamic testing results—4-point bending.

Sample	Extension	$F_{\min}\;\left[N\right]$	$F_{\max}\;\left[N\right]$	Recorded Cycles	Failure Mode
a	d=0mm	390.0	3900.0	7404	Fracture
b	d=80mm	290.0	2900.0	6734	Fracture
c	d=0mm	100.0	1000.0	1,000,000	No failure
d	d=80mm	100.0	1000.0	1,000,000	No failure

## Data Availability

The original contributions presented in the study are included in the article, further inquiries can be directed to the corresponding author.
